# Targeting CEA in Pancreas Cancer Xenografts with a Mutated scFv-Fc Antibody Fragment

**DOI:** 10.1186/2191-219X-1-24

**Published:** 2011-11-07

**Authors:** Mark D Girgis, Tove Olafsen, Vania Kenanova, Katelyn E McCabe, Anna M Wu, James S Tomlinson

**Affiliations:** 1 Department of Surgery, UCLA, 10833 LeConte Ave, Rm 54-140, Los Angeles, CA 90095, USA; 2 Crump Institute for Molecular Imaging, Department of Molecular and Medical Pharmacology, UCLA, Rm 4324E, CNSI, Bldg 114, 570 Westwood Pl, Los Angeles, CA 90095, USA; 3 Department of Surgery, Veterans Affairs, Greater Los Angeles, 11301 Wilshire Blvd, Bldg 500, Los Angeles, CA 90073, USA

**Keywords:** imaging, pancreas cancer, CEA, antibody

## Abstract

**Background:**

Sensitive antibody-based tumor targeting has the potential not only to image metastatic and micrometastatic disease, but also to be the basis of targeted therapy. The vast majority of pancreas cancers express carcinoembryonic antigen (CEA). Thus, we sought to evaluate the potential of CEA as a pancreatic cancer target utilizing a rapidly clearing engineered anti-CEA scFv-Fc antibody fragment with a mutation in the Fc region [anti-CEA scFv-Fc H310A].

**Methods:**

Immunohistochemistry (IHC) with the antibody fragment was used to confirm expression of CEA on human pancreas cancer specimens. *In vivo *tumor targeting was evaluated by tail vein injection of I^124^-labeled anti-CEA scFv-Fc(H310A) into mice harboring CEA-positive and -negative xenografts. MicroPET/CT imaging was performed at successive time intervals. Radioactivity in blood and tumor was measured after the last time point. Additionally, unlabeled anti-CEA scFv-Fc(H310A) was injected into CEA-positive tumor bearing mice and *ex vivo *IHC was performed to identify the presence of the antibody to define the microscopic intratumoral pattern of targeting.

**Results:**

Moderate to strong staining by IHC was noted on 84% of our human pancreatic cancer specimens and was comparable to staining of our xenografts. Pancreas xenograft imaging with the radiolabeled anti-CEA scFv-Fc(H310A) antibody demonstrated average tumor/blood ratios of 4.0. Immunolocalization demonstrated peripheral antibody fragment penetration of one to five cell diameters (0.75 to 1.5 μm).

**Conclusions:**

We characterized a preclinical xenograft model with respect to CEA expression that was comparable to human cases. We demonstrated that the anti-CEA scFv-Fc(H310A) antibody exhibited antigen-specific tumor targeting and shows promise as an imaging and potentially therapeutic agent.

## Introduction

Pancreatic cancer is one of the most lethal cancers as incidence approximates mortality [1]. Signs and symptoms that suggest pancreatic cancer are usually vague and occur late in the disease process. Because of this, most patients have metastatic disease at diagnosis resulting in an overall survival of 6% at 5 years [2]. Cure for pancreatic cancer currently hinges upon early diagnosis and surgical resection; however, only 10% to 20% of patients are eligible for surgery at diagnosis due to the presence of locally advanced cancer or metastatic disease [3]. Even still, this cohort of patients has poor survival due to the presence small foci of metastatic disease that is not detected by current imaging modalities. Given our current inability to detect the true burden of disease, pancreas cancer patients are routinely understaged and our local therapies are thus misguided. These data indicate the need to develop novel strategies to detect these small foci of disease for more accurate staging of pancreatic cancer so that we may apply our therapies appropriately.

**Figure 1:**
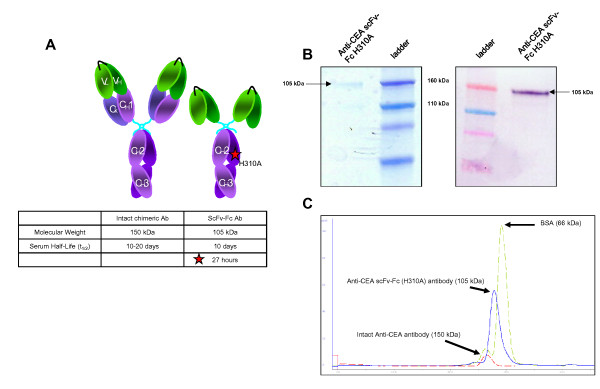
**A chimeric intact antibody and single-chain Fv-Fc (scFv-Fc) fragment**. **(a) **Schematic representation of a chimeric intact antibody and single-chain Fv-Fc (scFv-Fc) fragment. The table below the figure indicates the molecular weight and half-life of the antibodies. Also as shown, mutating the Fc region of an antibody at residue 310 from a histidine to an alanine will change the half-life significantly to only 27 h. **(b) **SDS-PAGE and Western blot of the anti-CEA scFv-Fc (H310A) antibody. The black arrow points to the purified antibody. **(c) **Size exclusion chromatography of intact CEA antibody, Anti-CEA scFv-Fc H310 antibody, and BSA. The peak of the scFv-Fc between the intact antibody and BSA confirms its intermediate size.

One such strategy to improve our ability to detect cancer is by using labeled antibodies targeting cancer-specific antigens. Antibodies offer high specificity for tumor antigens on the cell surface and thus can be used for positron emission tomography (PET) imaging once radiolabeled with a positron-emitting radionuclide (immunoPET). This offers great potential to achieve specific molecular imaging of cancer. Although very stable and specific, intact monoclonal antibodies are limited for imaging purposes by their extended serum half-life causing a high background signal. To circumvent this issue, recombinant, domain-deleted, antibodies with varying size and half-life can be engineered [4]. These recombinant antibodies possess similar antigen specificity as the parental intact antibody while exhibiting faster blood clearance. We have previously described the production of a chimeric anti-carcinoembryonic antigen (CEA) single-chain Fv-Fc (scFv-Fc) antibody fragment that contains a mutation in the Fc portion (histidine at position 310 to an alanine) [5]. This mutation was shown to reduce the serum half-life of the scFv-Fc fragment from 10 days to 27 h by preventing the interaction of the intact Fc region with the Brambell receptor (FcRN) responsible for diverting antibodies away from the degradation pathway in cellular lysosomes (Figure 1a).

CEA is a 180-kDa GPI-linked glycoprotein expressed on the cell surface of the normal adult colon at very low levels. However, during carcinogenesis, this oncofetal protein becomes much more highly expressed on the cell surface. Additionally, this protein can be shed into the circulation and measured as a serum tumor marker, reflective of the burden of disease [6]. High levels of CEA expression have been noted on a variety of gastrointestinal epithelial tumors. Adenocarcinoma of the pancreas is no exception, where increased CEA expression has been reported [6-9]. Here, we sought to investigate the potential of CEA as a tumor target of pancreas cancer utilizing our anti-CEA scFv-Fc H310A antibody fragment [5]. First, we validated CEA expression in our pancreas cancer xenograft models and in human pancreatic cancer specimens by performing immunohistochemistry (IHC) with our scFv-Fc (H310A) antibody fragment. We then evaluated our anti-CEA scFv-Fc (H310A) antibody fragment for *in vivo *antigen-specific tumor targeting of our xenograft models with microPET imaging. Lastly, we investigated the microscopic pattern of tumor targeting of the intravenously injected antibody fragment with *ex vivo *immunolocalization studies to detect the exact location of the fragment within the tumor, which may have important ramifications for development of antibody-based therapeutics.

## Materials and methods

### Production, purification, and characterization

Production, purification, and characterization of the anti-CEA scFv-Fc (H310A) antibody have previously been reported in detail [5]. Briefly, after gene assembly and site directed mutagenesis, 1 × 10^7 ^NS0 murine myeloma cells were transfected by electroporation with 40 μg of the pEE12 vector containing the anti-CEA scFv-Fc(H310A) construct and selected in glutamine-deficient DMEM/high modified media (JRH Biosciences, Lenexa, KS, USA) as previously described [5,10]. Cell culture supernatants were screened by enzyme-linked immunosorbent assay and Western blot for selection of high expressing clones followed by sequential protein purification with anion exchange and hydroxyapatite columns by fast performance liquid chromatography (FPLC) [5]. The final concentration of protein was determined by *A*_280 nm _using an extinction coefficient of *ε *= 1.4. Purity and size of the protein was determined by SDS/PAGE, Western blot, and size exclusion chromatography [5].

### Cell lines

NS0 mouse myeloma cells were maintained with DMEM/high modified media supplemented with 10% fetal bovine serum (FBS, Gemini Biosciences, West Sacramento, CA, USA), and 2 mM glutamine (Invitrogen, Carlsbad, CA, USA). The human pancreatic cancer cell lines, BxPC3, Capan-1, HPAF-II, and MiaPaca-2 were obtained from the American Type Culture Collection (ATCC, Manassas, VA, USA). RPMI-1640 medium, Iscove's Modified Dulbecco's medium, Eagle's Minimum Essential medium, and DMEM were used for BxPC3, Capan-1, HPAF-II, and MiaPaca-2 cells, respectively. All media was supplemented with 100 units of penicillin, 100 μg of streptomycin and 10% FBS. Only DMEM used for MiaPaca-2 cells were additionally supplemented with horse serum (2.5%).

### Antigen quantification

The relative expression of CEA was determined for each cell line by flow cytometry. For each cell line, 1 × 10^6 ^cells were harvested from culture and resuspended in 250 μl of phosphate-buffered saline/1% fetal bovine serum (PBS/1%FBS). Primary, intact mouse anti-CEA antibody (Abcam, Cambridge, MA, USA) was added in abundance (4 μg) and incubated for 1 h. The samples were centrifuged at 1000 g for 10 minutes, the supernatant was discarded, and the sample was resuspended in 250 μl of PBS/1% FBS. The secondary antibody, fluorescin isothiocyanate (FITC)-conjugated goat anti-mouse IgG (Fc specific) antibody (4 μg), was incubated with each sample for 1 h and was similarly washed and resuspended. Negative controls included samples with cells only and samples with cells and secondary antibody only. Quantitation of antigen expression for each cell line was performed using the DAKO Qifikit according to the manufacturer's instructions (DAKO, Carpinteria, CA, USA). Briefly, control beads coated with known amounts of antibody and mimicking defined antigen densities were incubated with FITC-conjugated goat anti-mouse IgG (Fc specific) antibody (DAKO) and evaluated by flow cytometry. A standard linear regression plot and equation was extrapolated from the mean fluorescence intensity (MFI) of the control beads. Samples of human pancreatic cancer cells were incubated with commercial intact mouse monoclonal anti-CEA antibody (Invitrogen) and FITC-conjugated anti-mouse IgG and detected by flow cytometry. The MFI of these samples were then applied to the extrapolated equation to determine the antibody binding capacity and thus, based on indirect immunofluorescence, the antigen density per cell. All experiments were performed in triplicate and averaged to provide reliable results.

### Immunohistochemistry

Human tissue specimens were provided by the Department of Pathology at University of California, Los Angeles (UCLA) Medical Center under an approved UCLA Institutional Review Board protocol. These specimens were evaluated by IHC for expression of CEA using the anti-CEA scFv-Fc(H310A) antibody fragment. Each paraffin embedded specimen was deparaffinized and incubated with the primary anti-CEA scFv-Fc (H310A) antibody fragment (1:50) for 1 h. Specimens were washed with PBS/1%Tween. Specimens were then incubated with the secondary mouse anti-human IgG (Fc specific) antibody (1:200) (Jackson Immunoresearch Laboratories, West Grove, PA, USA). After another wash, specimens were incubated with the tertiary horseradish peroxidase (HRP)-conjugated goat anti-mouse IgG (Fc specific) antibody (1:400) (DAKO). Negative control slides were only incubated with the secondary and tertiary antibodies.

### Radioiodination

Radioiodination with the positron-emitting isotope ^124^I was done by the Iodo-Gen method as described [5]. Labeling reactions (0.1 to 0.2 ml) typically contained 0.1 to 0.2 mg purified protein and 0.5 to 1 mCi Na^124^I (IBA Molecular, Dulles, VA, USA). Labeling efficiency was measured by instant thin layer chromatography (TLC) using the Tec-Control kit (Biodex Medical Systems, Shirley, NY, USA). Immunoreactivity was determined by incubating the radioiodinated anti-CEA scFv-Fc (H310A) antibody (≈100,000 cpm) with excess antigen-positive cells such that there was an abundance of antigen. After incubation and centrifugation, supernatant was collected and measured for the presence of radioactivity. The immunoreactive fraction was determined by use of the following equation: 1-(supernatant radioactivity/total radioactivity).

### Xenograft imaging and biodistribution studies

All animal handling was done under a protocol approved by the Chancellor's Animal Research Committee of the UCLA. Mouse xenografts were established in 8-week-old female nude mice (Charles River Laboratories, Wilmington, MA, USA). Three tumor models were developed with antigen-positive tumors on the left shoulder and antigen-negative tumors on the right shoulder so that each mouse served as its own control. Approximately 1 × 10^6 ^cells of an antigen-positive (BxPC3, HPAF-II, Capan-1) or -negative (MiaPaca-2) cancer cells were injected subcutaneously (s.c.) and allowed to grow for 10 to 14 days before imaging. Lugol's solution (0.5 ml per 100 ml water) was added to the drinking water 24 h prior to injection to block thyroid uptake of radioiodine. Also, gastric lavage with 1.5 mg of potassium perchlorate in 0.2 ml of PBS 30 min prior to tail vein injection was performed to block stomach uptake of radioiodine. Mice were injected with approximately 50 μg of ^124^I-anti-CEA scFv-Fc (H310A) antibody (specific activity of 2.40 μCi ± 0.005 μCi/μg) in PBS/1%FBS via the tail vein. At 4 and 20 h post-injection, the mice were anesthetized using 1.5% to 2% isoflurane, placed on the micro positron emission tomography (microPET) bed, and imaged with a Focus microPET scanner (Concorde Microsystems Inc., Knoxville, TN, USA). Acquisition time was 10 min. All images were reconstructed using a FBP algorithm and displayed by the AMIDE software package [11,12]. Selected animals were also imaged by micro computed tomography (microCT) with the resultant images coregistered with the microPET scans for anatomic reference. Following the last scanning time point, animals were euthanized; tumors and blood were harvested and weighed. Radioactive uptake of organs was counted in a gamma counter (Wizard 3″ 1480 Automatic Gamma Counter, Perkin-Elmer, Covina, CA) for biodistribution analysis. After decay correction, radioactive uptake in the tumor and blood was converted to percentage of injected dose per gram of tissue (%ID/g).

### Immunolocalization

IHC staining was also performed on paraffin-embedded sections of HPAF xenografts to evaluate for the presence of the intravenously injected anti-CEA scFv-Fc (H310A) antibody fragment. Mice were injected with 1 × 10^6 ^cells s.c. for xenograft creation. Once an appropriate size was achieved (approximately 0.5 cm), the mice were injected with 50 μg of anti-CEA scFv-Fc(H310A) antibody via the tail vein. After 20 h, mice were sacrificed and tumors were harvested, placed in a dry ice/2-butane bath and embedded in optimal cutting temperature solution. Frozen tumors were cut with a cryostat into 4-μm sections, placed on a glass slide, and fixed with 20% acetone for 20 min. After fixation, IHC staining was performed. All tumor specimen slides were incubated for 10 min with 3% hydrogen peroxide in methanol for blocking of endogenous peroxidase. Tumor specimen slides were incubated with the secondary mouse anti-human IgG (Fc specific) antibody (1:200) (Jackson Immunoresearch Laboratories) followed by the tertiary HRP-conjugated goat anti-mouse IgG (Fc specific) antibody (1:400) (DAKO). The positive control slides were incubated with intact mouse anti-CEA antibody (1:50) and HRP-conjugated goat anti-mouse IgG (Fc specific) antibody (1:400) (DAKO). Negative control slides were only incubated with the secondary and tertiary antibodies. Lastly, one slide was used for hematoxylin and eosin staining.

## Results

### Production, purification, and characterization

The anti-CEA scFv-Fc (H310A) antibody fragment was expressed in murine NS0 myeloma cells. Clones producing the highest amount of antibody by Western blot were selected for expansion. Protein was purified by FPLC from supernatant yielded an approximate purity of 98% by standard SDS-PAGE and Western blot (Figure 1b) [5]. Also, this scFv-Fc fragment had an elution time from a size exclusion chromatography column between that of the intact IgG (150 kDa) and bovine serum albumin (66 kDa) standards confirming its intermediate size of 105 kDa (Figure 1c) [5].

### Antigen quantification

**Figure 2:**
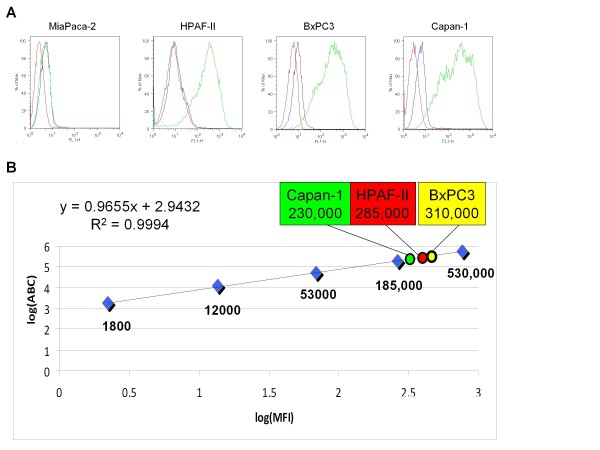
***In vitro *antigen quantification**. **(a) **Flow cytometry of each cell line tested for evaluation of CEA expression qualitatively and quantitatively. For each graph, the red curve corresponds to cells only, the blue curve to cells and secondary FITC-conjugated goat anti-mouse IgG (Fc specific) antibody, and the green curve to cells, primary mouse anti-CEA antibody, and secondary FITC-conjugated goat anti-mouse IgG (Fc specific) antibody. **(b) **Graph and linear regression equation of control beads used to determine antigen density per cell. The corresponding cell line antigen density is plotted and indicated on the graph.

CEA expression was determined for four different pancreatic cell lines (BxPC3, HPAF-II, Capan-1, and MiaPaca-1) using flow cytometry (Figure 2). MiaPaca-2 was the only human pancreatic cancer cell line tested that had no CEA expression and thus served as our negative control for experiments. Using the DAKO Qifikit, we quantified antigen expression by flow cytometry. CEA expression was approximately 230,000 (± 19,500), 285,000 (± 42,900), and 310,000 (± 45,000) antigens per cell for the Capan-1, HPAF-II, and BxPC3 cell lines, respectively. All studies were done in triplicate.

### Immunohistochemistry

**Figure 3:**
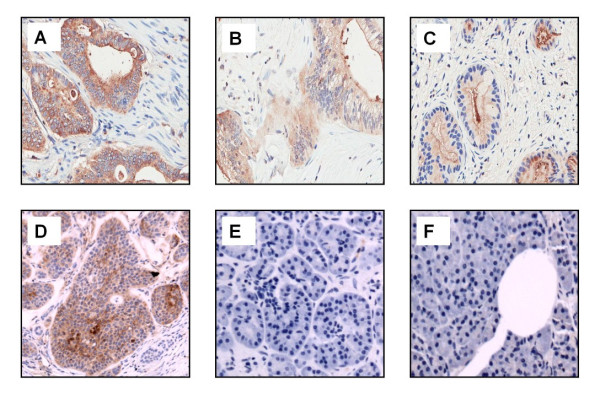
**Representative slides of IHC staining with anti-CEA scFv-Fc (H310A) antibody of different tissue specimens**. At ×40 magnification, **(a) **human pancreas cancer with strong staining, **(b) **human pancreas cancer with moderate staining, **(c) **human pancreas cancer with weak staining, **(d) **mouse pancreas cancer xenograft, **(e) **normal human pancreas, and **(f) **normal human liver.

Expression of CEA on human pancreas cancer specimens was evaluated by performing IHC utilizing the anti-CEA scFv-Fc (H310A) antibody fragment upon a tissue microarray containing 107 1-mm-tissue cores of pancreatic adenocarcinomas. Of the 107 cancer specimens, 90 demonstrated moderate to strong staining for CEA expression (Figure 3). Twelve specimens demonstrated weak expression and only four specimens showed no expression of CEA. IHC staining intensity was similar between the majority of human pancreas cancer specimens and mouse xenografts, both showing strong staining. Also, normal human liver and pancreas sections revealed no staining confirming low or no expression on normal tissues.

### Radioiodination, xenograft imaging, and biodistribution studies

**Figure 4:**
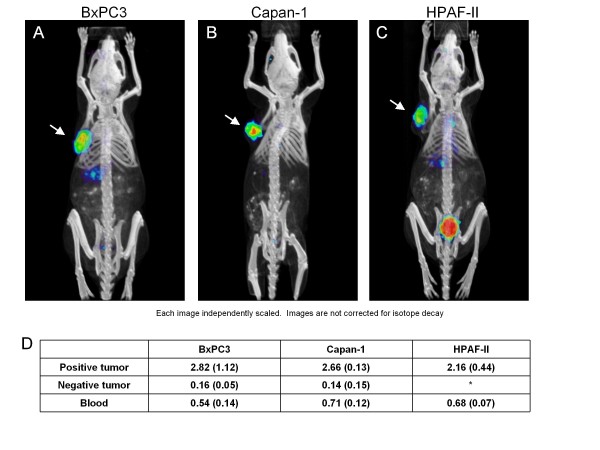
**MicroPET and MicroCT images of a representative mouse from each group at 20 h**. After tail vein injection showing targeting of each xenograft with the anti-CEA scFv-Fc (H310A) antibody fragment. Note CEA-positive xenografts (arrow) were not in the same plane as CEA-negative xenografts although present on all mice except HPAF tumor bearing mice. **(a) **BxPC3 tumor xenograft mouse, **(b) **Capan-1 tumor xenograft mouse, **(c) **HPAF-II tumor xenograft mouse. **(d) **Table with the corresponding measured radioactivity of each tissue. Values are represented as percent of injected dose per gram of tissue (%ID/g).

Radioiodination with ^124^I was performed with a labeling efficiency of 43%. Immunoreactivity of the labeled fraction was 83%. For animal studies, microPET/CT was employed to evaluate *in vivo *tumor targeting ability of the anti-CEA scFv-Fc (H310A) antibody fragment. Nude mice with a CEA-positive tumor (Capan-1, HPAF-II, BxPC3) and CEA-negative tumor (MiaPaca-2) were injected via the tail vein with approximately 50 μg of radiolabeled antibody fragment (120 μCi of radioactivity). Three mice per positive cell line were used for imaging purposes. Average tumor weight for all positive tumors was approximately 220 mg (range, 83 to 446 mg). Whole body microPET scans were obtained at 4 and 20 h post-injection. MicroCT was obtained at 20 h only. Figure 4 illustrates a representative image of a member of each animal group at 20 h. Images shown indicate specific uptake of the radiolabeled anti-CEA scFv-Fc (H310A) antibody fragment on the left shoulder of the mouse where positive xenografts were grown. There is little background activity visualized by microPET. The percent of injected dose per gram of tissue for positive tumor, negative tumor and blood for each of the animal groups to provide objective confirmation of the microPET images are also shown in Figure 4. Average tumor to blood ratios for Capan-1, HPAF-II, and BxPC3 were 3.7, 3.2, and 5.2, respectively. Average positive tumor to negative tumor ratios for Capan-1 and BxPC3 were 18.1 and 17.6, respectively. For the group of animals with HPAF-II tumors, the negative tumor was not identifiable upon imaging or necropsy; thus, no data is reported for the negative tumor. Biodistribution data for all other organs evaluated were not performed in this study as our group has previously published these results [5].

### Immunolocalization

**Figure 5:**
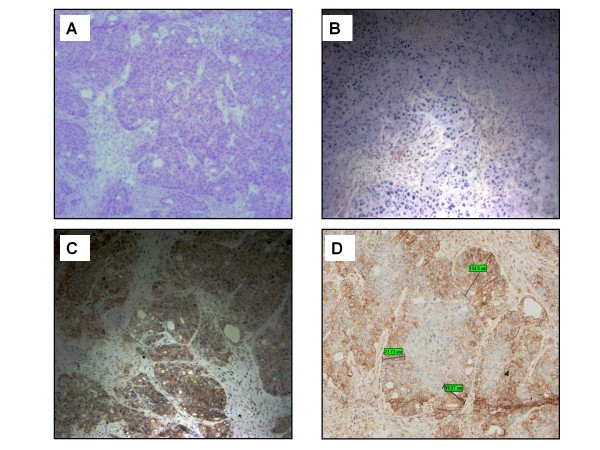
**Immunolocalization of anti-CEA scFv-Fc (H310A) antibody fragment after tail vein injection into HPAF-II tumor-bearing mice**. At ×20 magnification, **(a) **H&E-stained section, **(b) **negative control; slide incubated with only with secondary HRP-conjugated goat anti-mouse IgG (Fc specific) antibody, **(c) **positive control; slide incubated with primary mouse anti-CEA antibody and secondary HRP-conjugated goat anti-mouse IgG (Fc specific) antibody, **(d) **slide incubated with mouse anti-human IgG (Fc specific) antibody and HRP-conjugated goat anti-mouse IgG (Fc specific) antibody.

IHC staining was also performed on frozen tumor sections from mice harboring HPAF-II xenografts after tail vein injection of 50 μg of the unlabeled or "cold" anti-CEA scFv-Fc (H310A) antibody fragment and a 20-h *in vivo *incubation period. Sections were examined for the presence of the human Fc portion of the anti-CEA fragment. Intratumoral staining was largely localized to tumor cells at the periphery of the microtumor nodules surrounded by stroma and vessels (Figure 5). In comparison, the positive control slide showed membrane staining of all cancer cells regardless of location with respect to stroma and vessels as would be expected from an *ex vivo *application of the primary anti-CEA antibody. The negative control section exhibited no staining.

## Discussion

Targeting cancer with antibodies is a rapidly expanding field seeking to provide new technology for diagnosis and therapy. Considering molecular imaging applications such as immunoPET, intact antibodies are limited due to their extended serum persistence resulting in a high background signal. However, the growing knowledge of antibody interactions with FcRN receptors resulting in prolonged serum persistence have allowed for development of engineered antibody fragments possessing shorter half-lives while providing the same specific binding to their antigen. In such a way, these engineered antibodies can overcome the limitations of intact antibodies. Indeed, many recent studies have demonstrated that smaller size antibody fragments as well as decreased serum persistence are better imaging agents owing to their improved tumor penetration and rapid blood clearance [4,13-15]. Previously, our group produced and characterized the anti-CEA scFv-Fc (H310A) antibody fragment with a significantly reduced serum half-life (27 h) when compared to the intact antibody (> 10 days) [5]. With this antibody fragment, we sought to demonstrate the potential of CEA as a target in pancreas cancer and to investigate the utility of this fragment in antigen-specific targeting within our pancreas cancer models.

CEA serum levels have been used clinically for many years to diagnose, stage, and follow patients with colorectal cancer. Although CEA serum levels are not widely elevated in pancreatic cancer, this antigen is expressed on the cell surface of the vast majority of pancreatic cancers. Many reports of CEA on pancreas cancer specimens describe expression ranging from 70% to 98% [7,8,16]. In addition to showing high CEA expression on pancreatic cancer cell lines, Kaushal et al. demonstrated tumor targeting of a fluorophore-conjugated intact anti-CEA antibody in a xenograft model of pancreas cancer with the aim of developing an intraoperative imaging probe [16]. Given the reported prevalent expression of CEA in pancreas cancers, we attempted to investigate the immunoPET imaging potential of the anti-CEA scFv-Fc (H310A) antibody fragment in pancreas cancer xenograft models. First, we confirmed high levels of expression on Capan-1, HPAF-II, and BxPC3 cancer cell lines and no expression of CEA on the MiaPaca-2 cell line. Additionally, we found that CEA expression was very similar between the positive CEA cell lines ranging from 230,000 to 310,000 antigens per cell. Next, utilizing a tissue microarray we simultaneously evaluated 107 surgically resected human pancreas cancer specimens for CEA expression with IHC to validate previous reports and compare with our xenograft models. We found moderate to strong staining of CEA on 84% of specimens consistent with results described in the literature [7,8,16]. Moreover, we demonstrated similar staining intensity between our mouse pancreatic xenografts and strongly stained human pancreas cancer specimens. Based on these results, we were satisfied that CEA is abundantly expressed in the majority of pancreas cancers and thus a suitable target. Furthermore, our xenograft model recapitulates the human condition with respect to CEA expression.

Using our pancreatic cancer mouse xenograft model, we injected mice with ^124^I-labeled anti-CEA scFv-Fc (H310A) antibody and imaged at 4 and 20 h after injection with microPET/CT. Our microPET images at 4 h demonstrate quick targeting of the antibody fragment to all CEA-positive pancreatic tumors (data not shown). Moreover, microPET images at 20 h show persistence of signal at the site of the tumor with low blood background signal. Accordingly, biodistribution data at 20 h after injection provides objective confirmation of the microPET images. We were able to achieve positive tumor to negative tumor ratios greater than 17 demonstrating antigen-specific tumor targeting of the anti-CEA scFv-Fc (H310A) antibody fragment. Furthermore, a tumor to blood ratio of 4.0 at 20 h is evidence of the imaging benefit afforded by the decreased serum half-life of the fragment. Overall, these data are very supportive regarding the immunoPET imaging potential of the anti-CEA scFv-Fc (H310A) antibody fragment in pancreas cancer.

To assess the potential ability of converting an antibody-based imaging agent into a tumor-targeting therapeutic, we additionally wanted to define the microscopic pattern of tumor targeting of the anti-CEA scFv-Fc (H310A) antibody fragment in mice xenografts by performing "immunolocalization" studies. These studies provided confirmatory evidence of the microPET images demonstrating the physical presence of the anti-CEA scFv-Fc (H310A) antibody protein in the tumor sections. Furthermore, the intratumoral staining pattern demonstrated localization of antibody to the periphery of microscopic tumor nodules comprising the macroscopic tumor xenograft with antibody penetration approximately one- to five-cell-layers deep from the intervening stroma and vessels. Additionally, antibody tumor penetration models describe a number of factors including antigen density, antibody binding affinity, and antibody metabolism along with physical properties of the cancer tissue (e.g. tumor vascularity) as impacting antibody localization [17-21]. Depending on the cytotoxic bystander effect of the therapeutic modality associated with the antibody fragment, extensive tumor penetration may not be necessary [19,22,23]. With respect to radioimmunotherapy, radionuclides such as Yttrium-90 possessing a relatively long radiation range (path length > 1 mm) may supply a sufficient dose of cytotoxic radiation to the nuclei of cells in center or cold area of the tumor micronodules which are not directly bound by the antibody fragment-radionuclide conjugate [23]. Additionally, switching to a radiometal with shorter beta particle range might be more appropriate in considering treatment of smaller tumor deposits such as micrometastases. Although utilizing biodistribution data from using a radioiodine labeled antibody fragment to estimate biodistribution of a radiometal labeled fragment was not performed, one can imagine based on the immunohistochemical staining pattern that a radiolabeled engineered antibody with modest tumor penetration, as demonstrated in this study with the anti-CEA scFv-Fc (H310A) antibody, may have applications for radioimmunotherapy. Future studies should be directed at determining the appropriate radionuclide (e.g. alpha-emitter, long- or short-path-length beta-emitter) sufficient to provide the bystander effect without compromising surrounding tissues.

This work demonstrates the utility of the anti-CEA scFv-Fc (H310A) antibody fragment for imaging pancreas cancer with possible applications for therapy. We show the *in vivo *imaging potential and targeting capability of this antibody fragment in pancreatic cancer xenografts. Although CEA expression appears to be similar between our xenografts and the majority of human pancreas cancer specimens, data from xenograft models are limited secondary to lack of a competent immune system. Historically, the majority of murine monoclonal antibodies have failed to be translated to the clinical setting because of the human anti-mouse antibody (HAMA) response. This resulted in the advent of chimeric antibodies with murine variable regions (V_L _and V_h_) and human constant domains (C_H_2 and C_H_3) as well as humanized and fully human antibodies. Of note, the anti-CEA scFv-Fc (H310A) antibody fragment is a chimeric protein, which should decrease the incidence of the HAMA response, although it may still occur with repeated administration of the protein [24].

In summary, antigen-specific molecular imaging has the potential to provide a more accurate assessment of the tumor burden for pancreatic cancer patients. CEA is strongly expressed in the majority of pancreas cancers and thus is a potential target for antibody-based molecular imaging and therapy. Using the novel mutated anti-CEA scFv-Fc (H310A) antibody fragment with a reduced serum half-life, we demonstrated *in vivo *antigen-specific molecular imaging. Furthermore, we define the microscopic pattern of tumor targeting which may have implications regarding radioimmunotherapy. The versatility of this antibody construct, based on the presence or absence of an Fc domain mutation, provides for improved pharmacokinetics in both imaging and therapy making it a very attractive fragment for continued study and development.

## Competing interests

The authors declare that they have no competing interests.

## Authors' contributions

MG carried out immunoassays, biochemical characterization, functional characterization and drafted the manuscript. TO participated in animal studies and manuscript preparation. VK participated in design of the study and animal studies. KM participated in animals studies and biochemical characterization. AM participated in design of the study and manuscript preparation. JT performed animal studies, carried out immunoassays, conceived the study and helped prepare the manuscript. All authors read and approved the final manuscript.
